# Assembly and Comparative Analysis of the Complete Mitochondrial Genome of *Hippophae salicifolia*

**DOI:** 10.3390/biology14040448

**Published:** 2025-04-20

**Authors:** Zhefei Zeng, Chunmin Mao, Zhuo Shang, Ngawang Norbu, Ngawang Bonjor, Xiaoyan Jia, Wei Li, Wenju Zhang, Junwei Wang, La Qiong

**Affiliations:** 1 Key Laboratory of Biodiversity and Environment on the Qinghai-Tibetan Plateau, Ministry of Education, School of Ecology and Environment, Tibet University, Lhasa 850000, China; zengzhefei@126.com (Z.Z.);; 2 Yani Observation and Research Station for Wetland Ecosystem, Tibet University, Nyingchi 860000, China; 3 School of Geography and Ecotourism, Southwest Forestry University, Kunming 650224, China; 4 Ministry of Education Key Laboratory for Biodiversity Science and Ecological Engineering, School of Life Sciences, Institute of Biodiversity Science, Fudan University, Shanghai 200433, China

**Keywords:** *Hippophae salicifolia*, mitochondrial genome, repetitive sequences, RNA editing, phylogeny

## Abstract

This study reports the first complete mitochondrial genome assembly of *Hippophae salicifolia*, an ecologically and economically important plant endemic to the Himalayas. The 475,105 bp genome has a 44.80% GC content and an overall AT bias, comprising 74 genes (37 protein-coding, 31 tRNA, three rRNA, and three pseudogenes). We identified extensive repetitive elements, including 188 SSRs, 20 tandem repeats, and 455 dispersed repeats, and explored their potential roles in genome evolution. Codon usage analysis showed a bias for codons ending in A or U, while RNA editing analysis revealed 415 sites that mostly convert hydrophilic to hydrophobic amino acids. Phylogenetic and collinearity analyses clarified evolutionary relationships within *Hippophae* and uncovered genome rearrangements. In addition, extensive gene transfer was detected between the mitochondrial and chloroplast genomes. Ka/Ks and nucleotide diversity analyses indicate that most genes are under purifying selection, with some possibly undergoing positive selection. Overall, these findings enhance our understanding of the structural and evolutionary features of the *H. salicifolia* mitochondrial genome and provide valuable insights for the genetic improvement and conservation of *Hippophae* species.

## Simple Summary

In this study, we present the first fully assembled mitochondrial genome of *Hippophae salicifolia*, a Himalayan endemic. The circular genome spans 475,105 bp, has a GC content of 44.80%, and encodes 74 genes. It is strongly AT-biased and enriched in repetitive elements, including SSRs, tandem repeats, and dispersed repeats. Codon usage analysis reveals a marked preference for specific codons, likely shaped by the genome’s AT composition, tRNA availability, and translational efficiency. RNA editing events predominantly convert hydrophilic to more hydrophobic amino acids. Phylogenetic reconstruction supports the accepted relationships within *Hippophae* and uncovers extensive gene-order rearrangements in the mitochondrial genome. Remarkably, we detect large-scale gene transfer between the mitochondrion and chloroplast. Ka/Ks and nucleotide diversity metrics indicate that most mitochondrial genes are under purifying selection, although a few show signatures of positive selection. Collectively, these findings refine our understanding of plant mitochondrial architecture, evolution, and function, and provide a valuable resource for the genetic improvement and conservation of *Hippophae* species.

## 1. Introduction

Mitochondria are indispensable organelles in most eukaryotic cells, generating ATP through oxidative phosphorylation to provide energy for cellular activities [1,2]. Beyond energy metabolism, mitochondria are involved in critical physiological processes such as metabolic regulation, apoptosis, stress responses, and cell division, playing a vital role in plant growth and development [3]. Compared to animal mitochondria, plant mitochondrial genomes exhibit unique characteristics, such as larger genome sizes, complex gene arrangements, and frequent structural rearrangements and inversions [4,5]. These complexities arise from the evolutionary trajectory of plant mitochondrial genomes following endosymbiotic events, including frequent recombination of repetitive sequences, horizontal gene transfer within or between cells, and the insertion of exogenous DNA [5,6]. Such evolutionary events have led to significant structural and size differences between plant and animal mitochondrial genomes, resulting in highly diverse and dynamic features [5,7].

The sizes of plant mitochondrial genomes vary considerably, ranging from 42 kb (e.g., *Mesostigma viride*) to 11.3 Mb (e.g., *Silene conica*) [8,9]. This vast difference is primarily attributed to the frequent recombination of repetitive sequences and the insertion of foreign sequences [5,10]. Repetitive sequences not only influence genome size but also play a crucial role in the stability and diversity of genome structure, particularly in the formation of multichromosomal structures and subgenomes [10,11]. Despite the high variability in size and structure, plant mitochondrial genomes exhibit relatively low mutation rates, particularly when compared to nuclear and chloroplast genomes [12]. This low mutation rate confers unique advantages to mitochondrial genomes in plant phylogenetics and evolutionary studies, with their highly conserved protein-coding genes providing important insights into plant evolutionary history [12]. However, significant differences exist among species regarding mitochondrial gene numbers and gene order, even though functional genes have remained relatively stable over long-term evolution.

The complex structure of mitochondrial genomes, along with frequent fragment exchanges with chloroplast genomes, poses challenges for assembling plant mitochondrial genomes using traditional short-read sequencing technologies, often resulting in incomplete assemblies or errors [11,13]. Repetitive sequences and complex genome structures may cause gaps or mismatches during assembly, affecting the integrity and accuracy of the genome [14]. Owing to the progress in long-read sequencing tech like Oxford Nanopore and PacBio, scientists now have access to extended read lengths and broader coverage. This development greatly aids in the precise elucidation of the intricate structures of mitochondrial genomes [13,15]. The application of these technologies has propelled progress in plant mitochondrial genome research, uncovering details of genome rearrangements, repetitive sequences, and inter-organellar gene transfer phenomena.

Despite technological advancements offering new opportunities, research on plant mitochondrial genomes remains relatively underdeveloped, particularly in angiosperms with rich species diversity [11,13]. Currently, numerous plant chloroplast genomes have been sequenced and utilized for phylogenetic analyses [16], but mitochondrial genome data are still limited. This limitation hampers our in-depth understanding of the evolution of plant mitochondrial genomes, genetic diversity, and the molecular mechanisms underlying plant adaptation to different ecological environments. Consequently, thoroughly characterizing mitochondrial genomes across a broader range of species—especially those that are ecologically vital or economically valuable—remains essential. Such studies would not only enrich the plant mitochondrial genome database but also provide new insights into research on plant phylogenetics, biogeography, and adaptive evolution.

*Hippophae salicifolia* D. Don, belonging to the genus *Hippophae* in the family Elaeagnaceae, is a deciduous tree or shrub primarily distributed in the Himalayan region [17]. This species is highly adaptable, cold-resistant, and tolerant of poor soils, often growing along riverbanks, slopes, and in thickets, playing an important role in soil and water conservation and ecological protection [18]. Furthermore, *Hippophae* species are rich in vitamins, flavonoids, and essential fatty acids, with significant medicinal and economic value, widely used in the production of food, medicine, and cosmetics [19,20]. Its fruits and leaves are rich in antioxidants, exhibiting anti-inflammatory, antibacterial, and cardiovascular protective effects [19,21]. Currently, genomic studies on *H. salicifolia* have only completed the sequencing and analysis of its complete chloroplast genome [22], while its complete nuclear and mitochondrial genomes remain unresolved, limiting our understanding of the genetic characteristics and adaptive evolution of this species.

To address the gap in mitochondrial genome research of *H. salicifolia*, this study presents the first complete assembly and in-depth analysis of its mitochondrial genome. We thoroughly analyzed its gene composition, characteristics of repetitive sequences, codon usage bias, and RNA editing sites, revealing the basic features of the mitochondrial genome of *H. salicifolia*. By comparing with other *Hippophae* species, we conducted phylogenetic and synteny analyses to explore genome rearrangements, gene transfer, and evolutionary relationships. Additionally, using Ka/Ks ratios and nucleotide diversity analyses, we assessed the selective pressures experienced by mitochondrial genes during evolution. Our findings not only enrich the genomic data of *Hippophae* species but also provide valuable insights into understanding the evolutionary mechanisms of plant mitochondrial genomes and the adaptability of *H. salicifolia*.

## 2. Materials and Methods

### 2.1. Sample Collection and Sequencing

Fresh young leaves of female *H. salicifolia* plants were collected in Cuona County, Tibet Autonomous Region (109°36′33.48″ E, 30°21′40.52″ N). Voucher specimens have been deposited in the Herbarium of the College of Ecology and Environment, Tibet University (No.: Lq20240673). The use of plant materials in this study adheres to all local, national, and international guidelines and regulations for plant research. The collected leaves were immediately frozen in liquid nitrogen and stored at −80 °C upon returning to the laboratory until DNA extraction. Genomic DNA was extracted using a modified CTAB method [23]. The integrity of the extracted DNA was assessed by 1.0% agarose gel electrophoresis, and its purity and concentration were evaluated using a NanoDrop 2000 spectrophotometer (NanoDrop Technologies, Wilmington, DE, USA). DNA samples meeting quality standards were sent to Novogene Bioinformatics Technology Co., Ltd. (Beijing, China) for library construction. The library was prepared using the PacBio SMRTbell template preparation method, suitable for HiFi sequencing. Sequencing was performed on the PacBio Sequel II platform with a target sequencing depth of 30×. HiFi sequencing followed the standard operating procedures of Pacific Biosciences, generating high-accuracy, long-read sequencing data for subsequent mitochondrial genome assembly.

### 2.2. Mitochondrial Genome Assembly and Annotation

De novo assembly of the HiFi read data was performed using PMAT v1.5.4 [24]. The initial assembly was visualized and further processed using Bandage v0.8.1 [25]. Based on coverage depth analysis, redundant sequences—likely derived from chloroplast or nuclear genomes—were identified and removed within the software, ensuring the accuracy and reliability of the final assembly. For gene annotation, reference mitochondrial genome sequences from *H. rhamnoides*, *H. tibetana*, *H. gyantsensis*, and *Arabidopsis thaliana* were used. Protein-coding genes (PCGs) were annotated using the online tools GeSeq (https://chlorobox.mpimp-golm.mpg.de/geseq.html; accessed on 13 November 2024) [26] and PMGA [27], with homologous comparisons conducted using BLASTN v2.10.1 [28]. Transfer RNA (tRNA) genes were identified using tRNAscan-SE [29], with parameters set to mitochondrial tRNA mode. Ribosomal RNA (rRNA) genes were annotated by BLASTN v2.10.1 [28] using known mitochondrial rRNA sequences. To improve annotation accuracy, automatic results were manually corrected using Apollo v1.11.8 [30]. The final mitochondrial genome map was generated using OGDRAW v1.3.1 (https://chlorobox.mpimp-golm.mpg.de/OGDraw.html; accessed on 13 November 2024), which displayed the genome structure and gene distribution. Additionally, the chloroplast genome of *H. salicifolia* was assembled using Oatk v1.0 (https://github.com/c-zhou/oatk; accessed on 16 November 2024) based on the same HiFi sequencing data. Chloroplast genome annotation was performed using CPGAVAS2 (http://47.96.249.172:16019/analyzer/annotate; accessed on 17 November 2024) [31], with manual corrections applied. The final annotated mitochondrial and chloroplast genome sequences have been deposited in the GenBank database under accession numbers PQ653489 and PQ469773.

### 2.3. Repetitive Sequence Analysis

An investigation of repetitive sequences in the mitochondrial genome of *H. salicifolia* was performed, including Simple Sequence Repeats (SSRs), tandem repeats, and dispersed repeats. SSRs were detected using the MISA web tool (https://webblast.ipk-gatersleben.de/misa/; accessed on 21 November 2024) [32], with parameters set for repeat unit lengths of 1–6 nucleotides and minimum repeat numbers: 10 for mononucleotide repeats; 5 for dinucleotide repeats; and 4 for trinucleotide repeats and longer. Tandem repeats were identified using Tandem Repeats Finder v4.09 [33], with parameters set to match percentage ≥ 90%, indels and mismatches ≤ 10%, and repeat unit length ≥ 6 bp. Dispersed repeats were identified using BLASTN v2.10.1 [28] with parameters set to a word size of 7 and an E-value of 1 × 10^−5^. To avoid overlap between repeat types, previously identified tandem repeats were excluded during the search for dispersed repeats. The layout of repetitive sequences in the mitochondrial genome was displayed with Circos (http://circos.ca/software/download/; accessed on 22 November 2024).

### 2.4. Codon Usage Bias Analysis

Codon usage bias in the PCGs of the mitochondrial genomes of *H. salicifolia*, *H. rhamnoides*, *H. tibetana*, and *H. gyantsensis* was analyzed. PCG sequences for each species were extracted using PhyloSuite v1.23 [34], and Relative Synonymous Codon Usage (RSCU) values were calculated using MEGA v7.0.26 [35]. RSCU values were visualized using the Bioinformatics Cloud Platform (http://112.86.217.82:9919/#/; accessed on 23 November 2024) to compare codon usage differences among species.

### 2.5. RNA Editing Site Prediction

Potential C-to-U RNA editing sites in the PCGs of the mitochondrial genome of *H. salicifolia* were predicted using the PmtREP tool on the Bioinformatics Cloud Platform (http://112.86.217.82:9919/#/; accessed on 10 November 2024).

### 2.6. Analysis of Homologous Fragments Between Organelle Genomes

BLASTN v2.10.1 [28] was used to compare the mitochondrial and chloroplast genomes of *H. salicifolia* to explore sequence homology. The mitochondrial genome served as the query sequence and the chloroplast genome as the target. Parameters were set as follows: match percentage ≥ 70%; E-value ≤ 1 × 10^−5^; and homologous fragment length ≥ 30 bp. The distribution of homologous sequences was visualized using Circos.

### 2.7. Phylogenetic Analysis

To investigate the phylogenetic relationships of *H. salicifolia* at the mitochondrial genome level, all complete mitochondrial genomes within the order Rosales were downloaded from NCBI. In addition to the four species from Elaeagnaceae assembled in this study, one representative species was selected from each genus within the families Rosaceae, Cannabaceae, Ulmaceae, Rhamnaceae, and Moraceae, totaling 21 species, for which the NCBI accession numbers are listed in Table S1. *Leucaena trichandra* and *Delonix regia* from Fabaceae were used as outgroups. Conserved protein-coding genes common to all species were retrieved with PhyloSuite v1.23 [34] and aligned in MAFFT v7.310 [36]. The optimal nucleotide substitution model was determined using jModelTest v2.1.10 [37], based on the corrected Akaike Information Criterion (AICc), selecting the GTR+I+G model. A phylogenetic tree was constructed using the maximum likelihood method in IQ-TREE v1.6.12 [38], with 1000 bootstrap replicates to assess node support. The final phylogenetic tree was visualized using FigTree v1.4.4 (http://tree.bio.ed.ac.uk/software/figtree/; accessed on 13 December 2024).

### 2.8. Ka/Ks Ratio Evaluation

To evaluate the selective pressure on mitochondrial genes of *H. salicifolia*, 13 representative species within Rosales were selected based on the phylogenetic tree results. These include *H. tibetana* (PP712939), *H. gyantsensis* (NC_086921), *H. rhamnoides* (PQ309694; PQ309695), and *H. salicifolia* (PQ653489) from Elaeagnaceae; *Hemiptelea davidii* (MN061667) from Ulmaceae; *Humulus lupulus* (NC_086845) and *Cannabis sativa* (NC_029855) from Cannabaceae; *Ficus carica* (NC_077626) and *Morus alba* (PQ060099; PQ060100) from Moraceae; *Ziziphus jujuba* (NC_029809) and *Ventilago leiocarpa* (OQ165322; OQ165323; OQ165324) from Rhamnaceae; and *Prunus mira* (NC_065231); and *Geum urbanum* (NC_065221) from Rosaceae. Homologous PCGs in the mitochondrial genomes of *H. salicifolia* and other species were identified using BLASTN v2.10.1 [28]. Multiple sequence alignments of shared PCGs were performed using MAFFT v7.310 [36]. The nonsynonymous substitution rate (Ka), synonymous substitution rate (Ks), and their ratio (Ka/Ks) for each gene were calculated using the MLWL model in KaKs Calculator v2.0 [39]. The results were visualized using box plots generated with the ggplot2 package in R to evaluate the selective pressures experienced by genes during evolution.

### 2.9. Nucleotide Diversity (Pi) Analysis

Multiple sequence alignments of homologous PCGs from the 13 representative species were conducted using MAFFT v7.310 [36]. Nucleotide diversity indices (Pi) for each gene were calculated using DnaSP v5 [40] to assess the degree of genetic variation.

### 2.10. Comparative Analysis of Mitochondrial Genome Structures

To explore the structural conservation and rearrangement patterns of mitochondrial genomes in Rosales, comparative synteny analysis was performed on *H. salicifolia* and 12 representative species from other families. Pairwise comparisons of mitochondrial genome sequences were conducted using BLASTN v2.10.1 [28] with parameters set to word size 7, E-value threshold 1 × 10^−5^, and minimum alignment length 300 bp to reliably detect homologous regions. The BLAST results were input into MCScanX [41] to identify and classify syntenic blocks. Genome feature files (GFF3 format) were manually curated to retain only mitochondrial protein-coding genes and ORFs for annotation consistency. Finally, the multi-species synteny map was visualized using Circos.

## 3. Results

### 3.1. Characteristics of the Mitochondrial Genome of H. salicifolia

**Figure 1:**
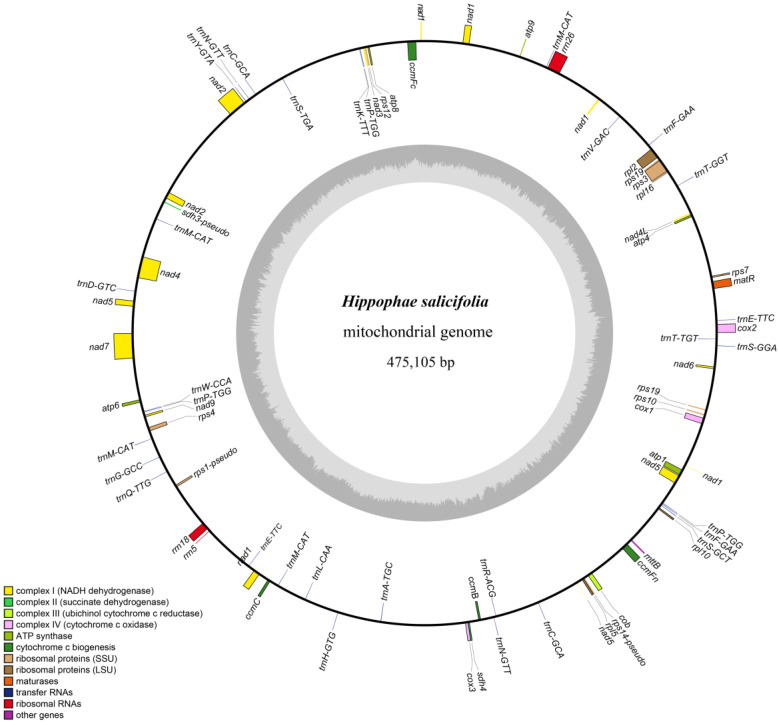
Map of the mitochondrial genome of *H. salicifolia*. Clockwise-transcribed coding genes are shown on the outer circle, while counterclockwise-transcribed coding genes are displayed on the inner circle. The innermost gray circle represents the GC content of the genome.

By analyzing the coverage differences of various fragments in the assembly draft of the mitochondrial genome of *H. salicifolia* (Figure S1), we successfully assembled a circular mitochondrial genome with a total length of 475,105 bp (Figure 1). The genome has a GC content of 44.80%, exhibiting an AT-biased nucleotide composition, a characteristic consistent with other plant mitochondrial genomes. Gene annotation revealed that the mitochondrial genome of *H. salicifolia* contains a total of 74 genes, including 37 PCGs, 31 tRNA genes, 3 rRNA genes, and 3 pseudogenes (Table S2). Of the genes identified, 25 unique ones form the core mitochondrial set, consisting of 5 ATP synthase, 9 NADH dehydrogenase, 4 cytochrome c biogenesis, 3 cytochrome c oxidase genes, plus 1 each of transport membrane protein, maturase, cytochrome b, and succinate dehydrogenase genes. Also found were 10 non-core mitochondrial genes: 4 large and 6 small subunit ribosomal protein genes.

Notably, some mitochondrial genes contain one or more introns. For example, *ccmFc*, *cox2*, *rpl2*, *rps3*, and *trnA-TGC* each contain one intron, while *nad1*, *nad4*, *nad5*, and *nad7* each contain four introns. Among all mitochondrial genes, eight genes were found to have multiple copies, mainly concentrated in tRNA genes. Specifically, *nad1* and *rps19* each have two copies; among the six multi-copy tRNA genes, *trnM-CAT* and *trnP-TGG* have the most copies, with four and three copies, respectively, while the remaining genes each have two copies (Table S2).

### 3.2. Analysis of Repetitive Sequences

**Figure 2:**
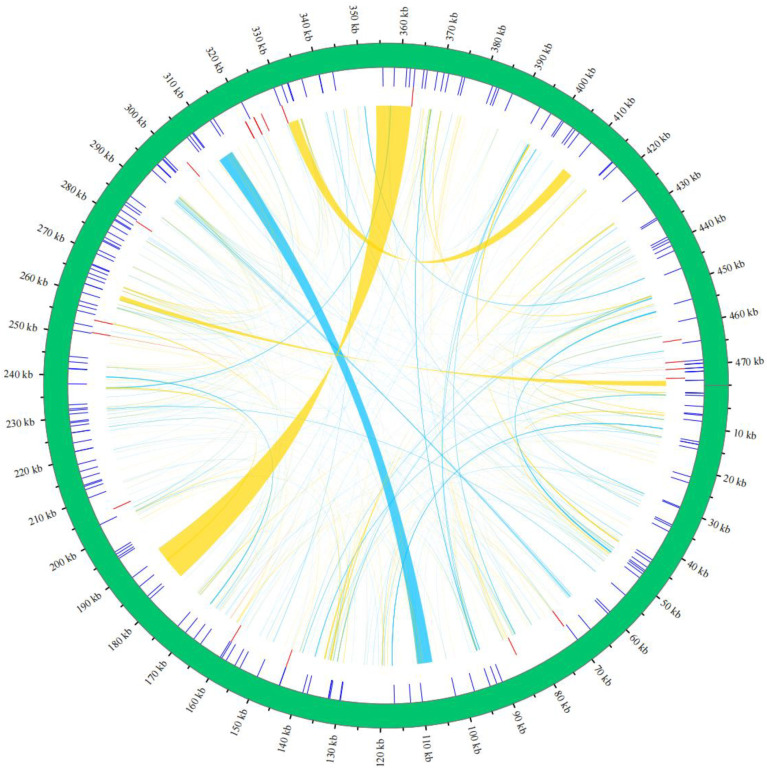
Distribution of repetitive sequences in the mitochondrial genome of *H. salicifolia*. The outermost circle represents the genomic coordinate range. SSRs and tandem repeats are indicated in blue and red, respectively, dispersed repeats are plotted in the innermost circle, with palindromic repeats shown in yellow and forward repeats in sky blue.

The mitochondrial genome of *H. salicifolia* contains abundant repetitive sequences, including 188 SSRs, 20 tandem repeats, and 455 dispersed repeats, which are widely distributed throughout the genome (Figure 2). Due to their characteristics of polymorphism, co-dominant inheritance, and widespread distribution, SSRs are commonly used as markers in studies of genetic diversity and evolution [42]. Among the identified SSRs, the types include mononucleotide, dinucleotide, trinucleotide, tetranucleotide, pentanucleotide, and hexanucleotide repeats (Table S3). Mononucleotide repeats are the most numerous, totaling 63 and accounting for 33.51% of all SSRs; followed by tetranucleotide repeats with 52 (27.66%); dinucleotide, trinucleotide, pentanucleotide, and hexanucleotide repeats account for 18.62%, 16.49%, 3.19%, and 0.53%, respectively. The diversity of these SSRs provides potential for developing species-specific markers.

Tandem repeats (satellite DNA), composed of core repeat units ranging from 1 to 200 bases arranged consecutively, are widely present in the genomes of eukaryotes and some prokaryotes [43,44]. Among the 20 tandem repeats identified in *H. salicifolia*, lengths range from 9 to 39 bases with a match percentage exceeding 72% (Table S4). The diversity of these tandem repeats may play important roles in gene regulation and genome structural stability. Dispersed repeats are also widely present in the genome. We identified a total of 455 dispersed repeat sequences with lengths ≥ 30 bp (Table S5), including 238 forward repeats, 213 palindromic repeats, and 4 reverse repeats. These dispersed repeats have a total length of 40,937 bp, accounting for 8.62% of the mitochondrial genome’s total length. In terms of length distribution, forward and palindromic repeats are most abundant in the 30–39 bp range, followed by the 40–49 bp range and repeats exceeding 100 bp (Figure S2). The longest forward repeat is 4105 bp, and the longest palindromic repeat reaches 9587 bp.

### 3.3. Codon Usage Analysis of Protein-Coding Genes

We analyzed the codon usage bias in the mitochondrial genomes of *H. salicifolia* and its close relatives *H. gyantsensis*, *H. rhamnoides*, and *H. tibetana*. Among these four species, *H. rhamnoides* has the highest number of codons at 10,360, followed by *H. tibetana* with 10,351, *H. salicifolia* with 10,181, and *H. gyantsensis* with 6143 (Table S6). Among all amino acids, arginine (Arg), leucine (Leu), and serine (Ser) have the highest usage frequencies, while methionine (Met) and tryptophan (Trp) have the lowest. This codon usage pattern is consistent with other plant mitochondrial genomes [4,45], indicating that the PCGs of *Hippophae* mitochondrial genomes exhibit relatively conserved characteristics during evolution. As shown in Table S3, most PCGs use ATG as the start codon, but *nad1* and *nad4L* genes use ACG as the start codon, which may be related to RNA editing processes. Regarding stop codons, TAA is the most commonly used, accounting for 54.29% of all stop codons, followed by TGA at 31.43%.

**Figure 3:**
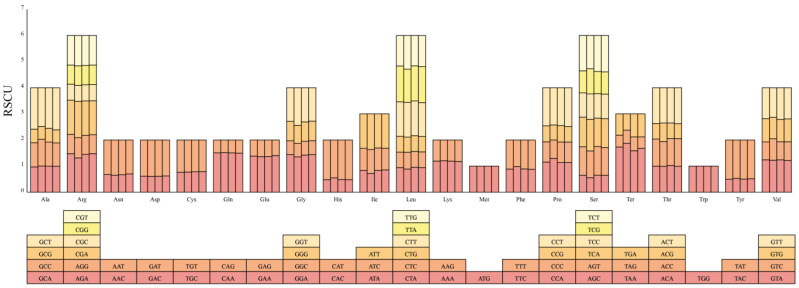
RSCU values in the mitochondrial genomes of four *Hippophae* species. The X-axis lists amino acids, and within each amino acid group, bars correspond to species in the following order: *H. salicifolia*, *H. gyantsensis*, *H. rhamnoides*, and *H. tibetana*. Bar colors uniquely denote individual codons, ensuring that the same color across species identifies the same codon.

To further explore codon usage bias, we calculated the RSCU values (Table S6). An RSCU value of 1 indicates no significant codon usage bias; values of less than 1 suggest the codon is used less frequently than its synonymous codons, while values greater than 1 indicate higher usage frequency. As shown in Figure 3, the mitochondrial PCGs of these four species generally exhibit usage preferences for specific codons. In *H. salicifolia*, *H. gyantsensis*, *H. rhamnoides*, and *H. tibetana*, the numbers of codons with RSCU values greater than 1 are 5946, 3721, 6173, and 6053, respectively, indicating these codons are used more frequently than their synonymous counterparts. Additionally, in all four species, most codons with RSCU values greater than 1 end with A or U, accounting for 94.00%, 93.50%, 94.20%, and 94.12% of the total, respectively. This phenomenon demonstrates a significant A/U preference in the highly used codons of these mitochondrial genomes.

### 3.4. RNA Editing Prediction

**Figure 4:**
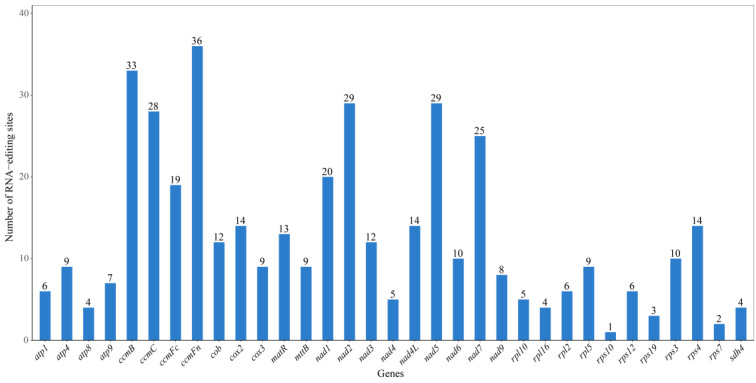
Distribution of RNA editing sites in protein-coding genes of the mitochondrial genome of *H. salicifolia*.

RNA editing refers to the process in eukaryotes where nucleotides are added, deleted, or substituted in the coding region of RNA transcripts [46,47]. In this study, we predicted 415 RNA editing sites across 31 protein-coding genes in the mitochondrial genome of *H. salicifolia* (Table S7, Figure 4). The distribution of RNA editing sites varies among different genes, ranging from one site (*rps10*) to 47 sites (*ccmFc*). After RNA editing, 43.37% of amino acids remain unchanged in hydrophobicity, 8.19% change from hydrophobic to hydrophilic, and 47.95% change from hydrophilic to hydrophobic. Additionally, 0.48% of amino acids are edited into stop codons (TGA) (Table S8).

We identified 30 types of codon transitions involving the conversion of 13 amino acids (Table S7). Among all codon transition types, the conversion from TCA to TTA is the most common, with 60 sites. The prediction results also show that leucine is the most frequently generated amino acid after RNA editing, accounting for 46.99% (195 sites). All RNA editing sites in the mitochondrial genome of *H. salicifolia* are of the C → U type; among them, 32.53% (135 sites) are located at the first nucleotide of the codon, 64.10% (266 sites) at the second nucleotide, and none at the third nucleotide. There are two special editing cases where both the first and second nucleotides of the codon are edited, leading to the conversion of proline (CCC, CCT) to phenylalanine (TTC, TTT), accounting for 3.37% (14 sites).

### 3.5. Homologous Fragment Analysis

**Figure 5:**
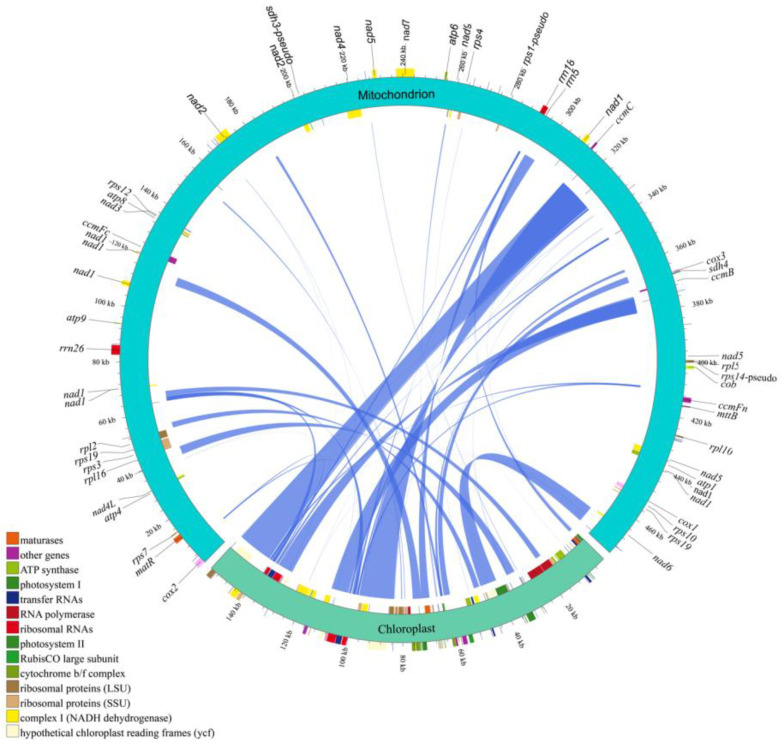
Homologous fragments between chloroplast and mitochondrial genome sequences in *H. salicifolia*. The chloroplast and mitochondrial sequences are shown, with homologous genes from the same complexes highlighted in matching colors. The lines in the center represent homologous sequences.

The mitochondrial and chloroplast genomes of *H. salicifolia* share 49 homologous fragments, covering 86,049 bp (18.11% of the mitochondrial genome), as shown in Figure 5 and Table S9. This percentage is much higher than in most other plants [45,48] and close to 17% in *H. tibetana* [4], indicating active gene transfer between organelles in the *Hippophae* genus. Among these fragments, 19 complete chloroplast protein-coding genes (e.g., *cemA*, *petA*, *petB*, *psbC*, *psbD*, *psbE*, *psbF*, *psbH*, *psbJ*, *psbL*, *psbN*, *psbT*, *rpl23*, *rps4*, *rps7*, *ycf1*, *ycf2*, *ndhB*, *ndhJ*), 15 tRNA genes (e.g., *trnD-GUC*, *trnF-GAA*, *trnH-GUG*), 4 rRNA genes (*rrn5S*, *rrn4.5S*, *rrn23*S, *rrn16S*), and many partial genes and intergenic regions were found. These results shed light on *H. salicifolia*’s genomic evolution and organelar genetic exchange.

### 3.6. Phylogenetic Relationships

**Figure 6:**
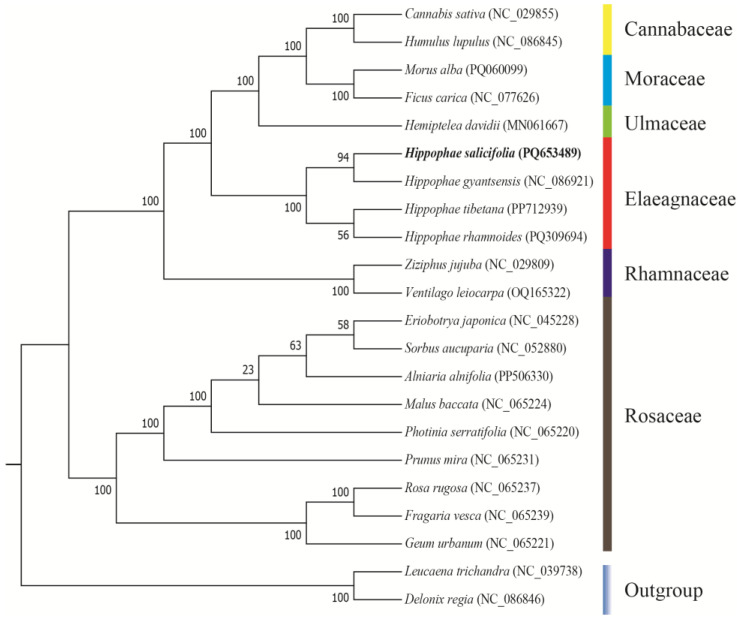
Phylogenetic tree derived from the mitochondrial protein-coding genes of 13 species. Bootstrap support values for each node are presented as percentages based on 1000 replicates. Different families are indicated by varying colors, with *H. salicifolia* highlighted in bold.

To investigate the evolutionary position of the *H. salicifolia* mitochondrial genome within the genus *Hippophae* and the broader plant phylogeny, we performed a phylogenetic analysis based on the DNA sequences of 30 shared mitochondrial PCGs from 22 species (Figure 6). The results revealed that the four *Hippophae* species clustered into two monophyletic groups: *H. salicifolia* and *H. gyantsensis* formed one clade, while *H. tibetana* and *H. rhamnoides* formed another. These two clades are sister groups, supporting the genetic differentiation and phylogenetic relationships within the genus.

This result aligns with the phylogeny constructed by Jia & Bartish (2018) based on nuclear gene fragments, further validating the utility of mitochondrial genomes in resolving plant evolutionary relationships [49]. Additionally, the phylogenetic tree showed that the Elaeagnaceae family clusters with species from Rhamnaceae, Ulmaceae, Moraceae, and Cannabaceae into a larger evolutionary branch, suggesting close relationships among these families. This observation is consistent with the latest Angiosperm Phylogeny Group (APG) classification system [50], supporting current perspectives in plant taxonomy.

### 3.7. Ka/Ks Ratio Analysis

**Figure 7:**
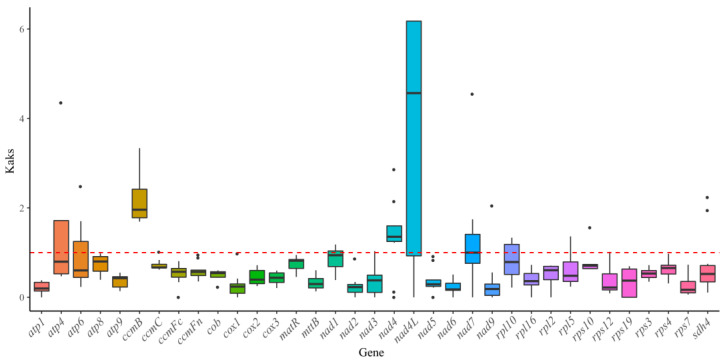
Boxplots showing pairwise Ka/Ks ratios among shared mitochondrial genome genes across 13 Rosales species. The red dashed line indicates the neutral expectation of Ka/Ks = 1. Genes with median Ka/Ks values above this line may be under positive selection, while those below are likely evolving under purifying selection.

To assess the selective pressures on the mitochondrial PCGs of *H. salicifolia* during evolution, we calculated the nonsynonymous (Ka) and synonymous (Ks) substitution rate ratios (Ka/Ks) between *H. salicifolia* and closely related species. The Ka/Ks ratio is a critical indicator for evaluating evolutionary rates and selection pressures on protein-coding genes. A Ka/Ks ratio of 1 indicates neutral evolution; a ratio less than 1 (Ka/Ks < 1) suggests purifying selection, indicating that functionally important genes are under conservative selective pressure; and a ratio greater than 1 (Ka/Ks > 1) points to positive selection, implying adaptive evolution [51]. The analysis (Figure 7) showed that most PCGs exhibited Ka/Ks ratios significantly lower than 1, indicating strong purifying selection and high functional conservation. Notably, genes such as *atp1*, *atp9*, *cox1*, *nad2*, *nad6*, and *rps7* displayed extremely low Ka/Ks ratios, suggesting their essential roles in maintaining mitochondrial function. In contrast, genes like *ccmB*, *nad4L*, and *nad4* had Ka/Ks ratios greater than 1, indicating potential positive selection, possibly associated with ecological adaptation. Some genes, including *atp4*, *atp6*, *nad1*, *nad*7, and *rpl10*, exhibited Ka/Ks ratios near or slightly above 1, indicating neutral evolution or weak positive selection.

### 3.8. Nucleotide Diversity Analysis

**Figure 8:**
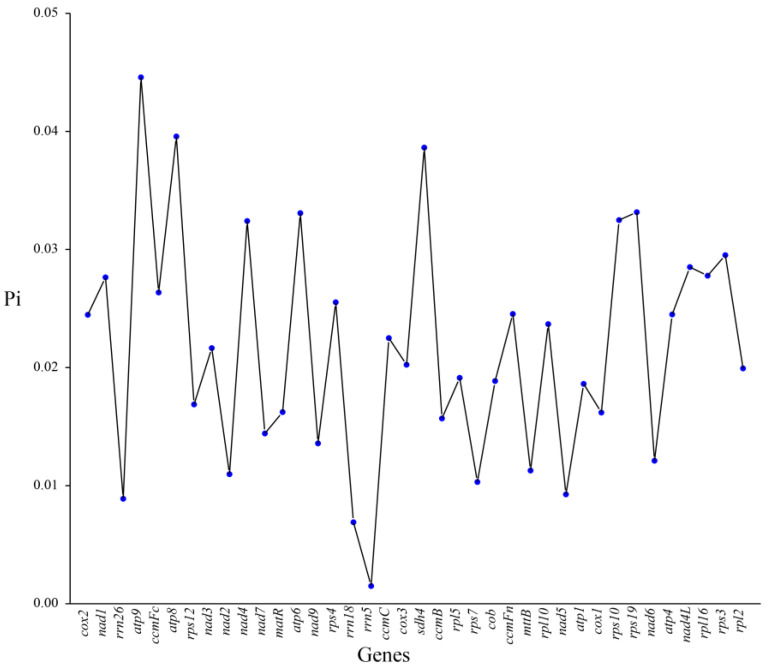
Nucleotide variability (Pi) across genes in the mitochondrial genome of *H. salicifolia*.

Nucleotide diversity (Pi) is a key metric for assessing genetic variation among species, commonly used in population genetics and evolutionary biology [52,53]. To evaluate genetic variation in the mitochondrial genomes of *H. salicifolia* and its close relatives, we analyzed nucleotide diversity in 36 PCGs and 3 rRNA genes across *H. salicifolia* and five related species. The results showed that most genes had Pi values below 0.04, suggesting high conservation, few mutations, and stable functions among species (Figure 8). This supports the concept of relative conservation in plant mitochondrial genomes during evolution.

However, a few genes, such as *atp9*, *atp8*, and *sdh4*, exhibited relatively high diversity, with Pi values of 0.04458, 0.03957, and 0.03863, respectively. This may reflect accelerated evolutionary rates in these genes within specific species, potentially due to adaptive evolution or functional differentiation. Additionally, genes like *nad5* and *rps7* had low Pi values (<0.01), indicating high conservation among Saxifragales species and suggesting they play key roles in maintaining essential mitochondrial functions, under strong purifying selection. For the three rRNA genes, especially *rrn5*, the extreme conservation (Pi = 0.0015) reflects their structural and functional importance, with minimal variation across species.

### 3.9. Collinearity Analysis

**Figure 9:**
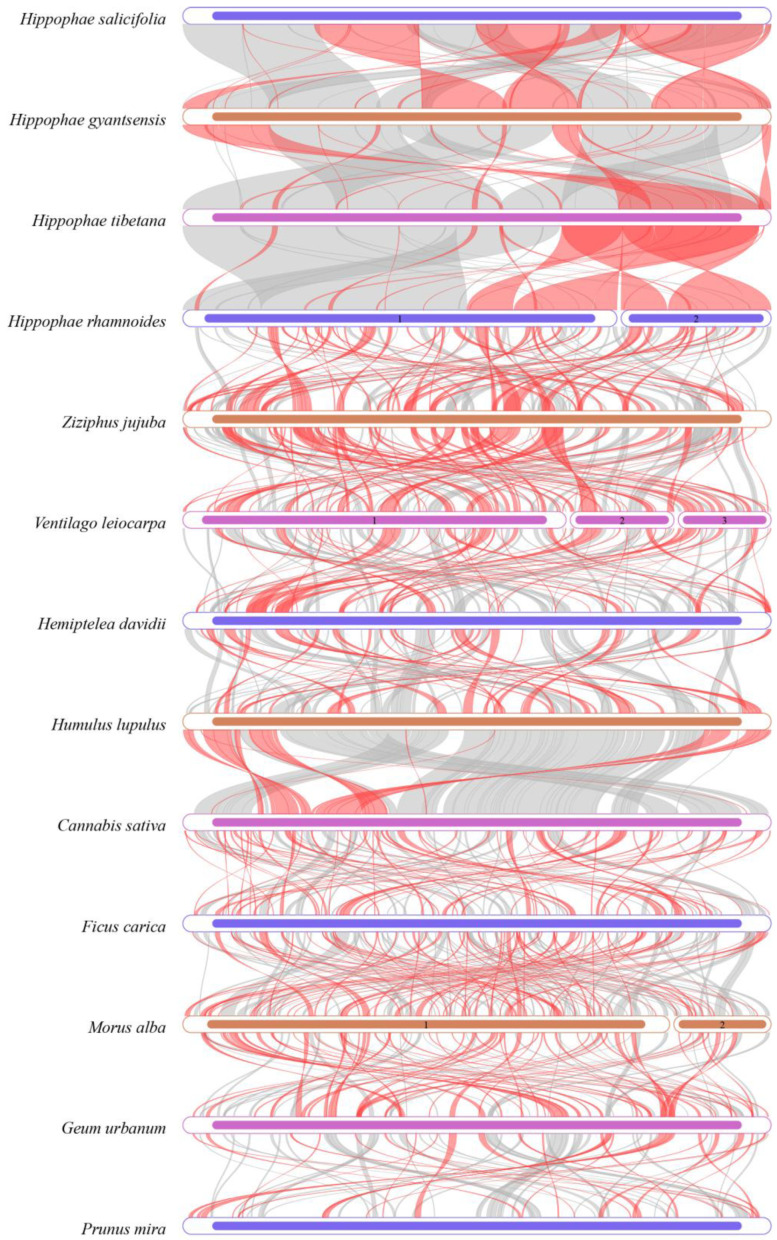
Multiple synteny plot comparing the mitochondrial genome of *H. salicifolia* with those of closely related species. Each row represents a genome, and the connecting lines indicate regions of homology. Red arcs denote inverted regions, while gray arcs indicate regions of higher homology.

To explore the structural differences and evolutionary relationships between the mitochondrial genome of *H. salicifolia* and other Rosales species, we conducted pairwise collinearity analyses. The results showed that the four *Hippophae* species shared the most homologous sequences, reflecting their close phylogenetic relationships. However, significant differences in gene arrangement were observed among their mitochondrial genomes, indicating extensive genomic rearrangements (Figure 9). These rearrangements included inversions in gene order, as well as insertions or deletions of gene fragments, likely driven by the presence of repeat sequences, intergenic recombination, and exogenous DNA insertions [5]. Such structural changes may play critical roles in species adaptation and evolution.

In comparison to other Rosales species, *Hippophae* species exhibited a higher frequency of genomic rearrangements and more complex mitochondrial genome structures. This suggests that significant structural changes have occurred during the evolution of *Hippophae* mitochondrial genomes, potentially related to ecological adaptations and evolutionary histories specific to this genus.

## 4. Discussion

### 4.1. Characterization of the H. salicifolia Mitogenome

This study successfully assembled and annotated the mitochondrial genome of *H. salicifolia* for the first time, filling a significant gap in mitochondrial genome research for this species. The results show that the genome is circular, with a length of 475,105 bp and a GC content of 44.80%, exhibiting a clear AT bias. These genomic characteristics are comparable to those of the closely related species *H. tibetana* [4], suggesting a high degree of conservation in mitochondrial genome size and GC content within this genus. Additionally, the observed AT-rich composition is a ubiquitous feature across plant mitochondrial genomes, underscoring its role as a common feature of base composition in these organelles [4,52].

Genome annotation revealed that the mitochondrial genome of *H. salicifolia* contains 74 genes, including 37 PCGs, 31 tRNA genes, 3 rRNA genes, and 3 pseudogenes. Among these, 25 core mitochondrial genes—such as those encoding ATP synthase, NADH dehydrogenase, cytochrome c biogenesis enzymes, and cytochrome c oxidase—are crucial for maintaining mitochondrial function and are highly conserved in plants, emphasizing their essential roles in energy metabolism and the respiratory chain [11]. The gene number and composition are consistent with those of *H. tibetana* [4], reflecting the conservation of mitochondrial genomes within this genus.

Notably, several genes contain introns. For example, *ccmFc*, *cox2*, *rpl2*, *rps*3, and *trnA-TGC* each contain one intron, while *nad1*, *nad4*, *nad5*, and *nad7* each contain four introns. The presence of introns may affect gene splicing and expression, thereby influencing mitochondrial function and efficiency [54]. Additionally, we found eight multi-copy genes, mostly among tRNA genes, with *trnM-CAT* and *trnP-TGG* having the highest copy numbers of four and three, respectively. Multi-copy tRNA genes may be related to genome recombination, replication mechanisms, and adaptive evolution. This phenomenon is relatively common in plant mitochondrial genomes, as reported in *A. thaliana* [55] and *Triticum aestivum* [56]. These multi-copy tRNA genes may increase tRNA availability to meet the demand for mitochondrial protein synthesis during rapid growth or under environmental stress [57].

### 4.2. The Role of Repetitive Sequences in Genome Structure and Evolution

Repetitive sequences play a key role in the structural variation and evolution of plant mitochondrial genomes, not only promoting genome rearrangements but also potentially affecting genome size through insertion or duplication [11,13]. In the mitochondrial genome of *H. salicifolia*, we identified abundant repetitive sequences, including 188 SSRs, 20 tandem repeats, and 455 dispersed repeats, which are widely distributed throughout the genome. These repetitive sequences probably add to the structural complexity of the *H. salicifolia* mitochondrial genome.

Among the SSRs, mononucleotide repeats are the most abundant, accounting for 33.51%, followed by tetranucleotide repeats at 27.66%. This distribution pattern is consistent with other plant mitochondrial genomes [4,48,52]. Due to their high polymorphism and widespread distribution, SSRs are often used as molecular markers in genetic diversity and evolutionary studies [42]. The diversity of SSRs in *H. salicifolia* provides a basis for developing species-specific molecular markers, aiding in population genetics and phylogenetic research.

Tandem repeats are also significantly present in the mitochondrial genome of *H. salicifolia*, with lengths ranging from 9 to 39 bp. Tandem repeats are believed to play important roles in gene regulation, gene expression, and genome structural stability. They may affect the three-dimensional structure of DNA, regulate gene transcription activity, and participate in DNA replication and repair processes [43,44].

The identified 455 dispersed repeats of lengths ≥30 bp exhibit diversity in number and length. Particularly, longer dispersed repeats may promote genome recombination and rearrangement, causing changes in gene order and structural variation, affecting gene function and expression, and providing a genetic basis for plant evolution and adaptation [11,58]. In the mitochondrial genome of *H. salicifolia*, the longest forward repeat and palindromic repeat reach lengths of 4105 bp and 9587 bp, respectively. Although long repeat sequences are relatively rare in plant mitochondrial genomes, their presence is often associated with genome instability and complex recombination events. They may become hotspots for genome rearrangement, increasing the complexity and diversity of mitochondrial genomes [58].

### 4.3. Codon Usage Bias and Adaptive Evolution

By analyzing codon usage bias in the mitochondrial PCGs of *H. salicifolia* and its close relatives, we found that arginine (Arg), leucine (Leu), and serine (Ser) have the highest codon usage frequencies, while methionine (Met) and tryptophan (Trp) have the lowest. This amino acid usage frequency distribution is consistent with other plant mitochondrial genomes [4,48,52], indicating that PCGs are conserved during evolution. Most PCGs use ATG as the start codon, but the nad1 and nad4L genes use ACG, which may be related to RNA editing. RNA editing is widespread in plant mitochondria, often leading to the formation or alteration of start codons, affecting gene expression and function [47].

RSCU analysis shows that the PCGs of these four species exhibit preferences for specific codons. Codons with RSCU values greater than 1 mainly end with A or U, accounting for approximately 94.00%. This preference may be related to the AT bias of the mitochondrial genome, reflecting the influence of base composition on codon usage. Codon usage bias may be influenced by factors such as mutation pressure, natural selection, and gene expression levels [4]. In plants, it is associated with tRNA abundance, translation efficiency, and adaptive evolution. Preferentially using codons ending with A or U may improve translation efficiency, affecting the speed and accuracy of protein synthesis [59].

### 4.4. Characteristics and Functional Significance of RNA Editing

RNA editing is a crucial post-transcriptional regulatory mechanism in plant mitochondrial genomes, altering genetic information by modifying mRNA bases and influencing gene expression and protein function [47]. We predicted 415 RNA editing sites in the mitochondrial genome of *H*. salicifolia, distributed across 31 protein-coding genes, with the number of edits ranging from 1 (rps10) to 47 (ccmFc).

RNA editing primarily leads to amino acid changes from hydrophilic to hydrophobic (47.95%), which may influence protein folding and stability, thereby enhancing their functional activity. Additionally, 0.48% of editing events generated stop codons, potentially causing premature protein termination and affecting gene function. A total of 30 types of codon conversions were identified, involving 13 amino acid changes. Among these, the most common conversion was from TCA to TTA (60 sites), resulting in the substitution of serine with leucine. Leucine accounted for 46.99% of the edited amino acids, consistent with findings in other plants [48,52]. As a hydrophobic amino acid, leucine is often located in the transmembrane regions of membrane proteins; thus, RNA editing may regulate the function of mitochondrial membrane proteins [47,60].

All editing events were of the C-to-U type, predominantly occurring at the second (64.10%) and first (32.53%) positions of the codon, with no editing observed at the third position. This aligns with characteristics observed in other plant mitochondria [4,48]. Notably, two editing events involved simultaneous changes at both the first and second codon positions, leading to the conversion of proline to phenylalanine. Such dual-site editing is relatively rare in plant mitochondria and may have special functional significance.

### 4.5. Phylogenetic Relationships, Collinearity Analysis, and Organelle Gene Transfer

Based on the mitochondrial protein-coding gene sequences of *H. salicifolia* and its closely related species, we constructed a phylogenetic tree to explore the evolutionary relationships within the genus *Hippophae*. The results showed that the four *Hippophae* species formed two monophyletic clades: *H. salicifolia* clustered with *H. gyantsensis*, while *H. tibetana* grouped with *H. rhamnoides*. These two clades are sister groups, supporting genetic differentiation within the genus. This finding is consistent with the phylogenetic analysis based on nuclear and chloroplast gene fragments by Jia & Bartish (2018) [49], verifying the reliability of mitochondrial genomes in revealing plant evolutionary relationships.

Utilizing the phylogenetic relationships, we selected representative *Hippophae* species for mitochondrial genome collinearity analysis. The results revealed that the four species share numerous homologous sequences, reflecting their close genetic relationships. However, significant differences in gene order were observed in their mitochondrial genomes, demonstrating extensive genome rearrangement events. These rearrangements include inversions of gene order and insertions or deletions of fragments, potentially caused by repeat sequences, intergenic recombination, or insertion of foreign DNA [5,6]. Plant mitochondrial genomes are known for their structural diversity and dynamics, with genome rearrangement being one of their key features [1,11]. Repeat sequences, especially long repeats, may promote non-homologous recombination, leading to structural changes in the genome [5].

Further studies revealed frequent gene transfer between the mitochondrial and chloroplast genomes of *H. salicifolia*, highlighting its genetic complexity. We identified 49 homologous fragments between the two genomes, covering 86,049 bp, which accounts for 18.11% of the mitochondrial genome. This percentage is far higher than in most plants [48,52] and matches the 17% in *H. tibetana* [4], indicating active gene flow between cellular compartments in this genus. Inter-organellar DNA transfer, especially from chloroplast to mitochondrion, is widespread in plants and is believed to play a significant role in genome evolution and functional diversity [11,13]. In the mitochondrial genome of *H. salicifolia*, we identified 19 complete chloroplast protein-coding genes, 15 tRNA genes, and 4 rRNA genes. The presence of these genes may enhance mitochondrial function or provide additional genetic material, aiding in environmental adaptation.

Additionally, phylogenetic analysis showed that species of the Elaeagnaceae family cluster with those from Rhamnaceae, Ulmaceae, Moraceae, and Cannabaceae into a large evolutionary branch, consistent with the latest Angiosperm Phylogeny Group (APG) classification, supporting current botanical taxonomy viewpoints [50]. This suggests a close phylogenetic relationship between Elaeagnaceae and these families, possibly sharing a common ancestor or similar evolutionary paths. Phylogenetic analysis based on mitochondrial genomes exhibits high resolution in elucidating inter-family and inter-genus relationships, likely due to the rich genetic information provided by mitochondrial genomes and their lower susceptibility to horizontal gene transfer and recombination [11]. However, combining data from nuclear and chloroplast genomes would be more effective for a comprehensive evolutionary picture.

### 4.6. Ka/Ks Ratio and Nucleotide Diversity Analyses

Ka/Ks ratio and Pi analyses are crucial tools for understanding the evolution of plant mitochondrial genes. We calculated the Ka/Ks ratios between *H. salicifolia* and closely related species to assess the selective pressures on mitochondrial protein-coding genes during evolution. The results showed that most PCGs had Ka/Ks ratios significantly less than 1, indicating strong purifying selection and high functional conservation. For instance, genes such as *atp1*, *atp9*, *cox1*, *nad2*, *nad6*, and *rps7* exhibited extremely low Ka/Ks ratios, suggesting their essential roles in maintaining basic mitochondrial functions [51]. However, genes such as *ccmB*, *nad4L*, and *nad4* had Ka/Ks ratios greater than 1, indicating that they may have undergone positive selection, potentially related to ecological adaptation and functional changes [51]. Additionally, genes like *atp4*, *atp6*, *nad1*, *nad7*, and *rpl10* had Ka/Ks ratios close to or slightly above 1, implying they may have experienced neutral evolution or weak positive selection.

Pi analysis further supports these findings. Most genes had Pi values below 0.04, indicating high conservation among species. However, a few genes such as *atp9*, *atp8*, and *sdh4* displayed higher diversity, with Pi values of 0.04458, 0.03957, and 0.03863, respectively, potentially associated with adaptive evolution or functional differentiation [52]. In contrast, genes like *nad5* and *rps7* had lower Pi values (<0.01), reflecting strong purifying selection. Moreover, the three rRNA genes, especially *rrn5*, showed extremely high conservation (Pi = 0.0015), emphasizing the importance of rRNA genes in protein synthesis and mitochondrial function.

Combining the Ka/Ks ratio and Pi analyses, we infer that most genes in the mitochondrial genome of *H. salicifolia* are under purifying selection, maintaining functional stability during evolution. However, certain genes may have undergone positive selection or neutral evolution, possibly related to ecological adaptation and functional differentiation. These findings provide new insights into the adaptive evolution of *Hippophae* species and enrich the study of evolutionary dynamics in plant mitochondrial genomes.

## 5. Conclusions

This study provides the first complete assembly and detailed analysis of the mitochondrial genome of *H. salicifolia*. The genome, a circular structure of 475,105 bp, exhibits a significant AT bias and shares structural and gene composition features with other *Hippophae* species, highlighting the conservation of mitochondrial genomes in the genus. We identified abundant repetitive sequences, including SSRs, tandem repeats, and dispersed repeats, which contribute to genomic complexity and evolution. Codon usage analysis revealed a preference for specific codons, likely influenced by the genome’s AT content, tRNA abundance, and translation efficiency. RNA editing predominantly causes hydrophilic-to-hydrophobic amino acid changes, potentially affecting protein function and stability. Phylogenetic analysis confirmed the relationships among *Hippophae* species, while collinearity analysis revealed significant gene-order rearrangements, underscoring the role of genome restructuring in evolution. Notably, we observed extensive gene transfer between the mitochondrial and chloroplast genomes of *H. salicifolia*, highlighting complex interactions between organelles. Ka/Ks ratio and nucleotide diversity analyses showed that most mitochondrial genes are under purifying selection, with some undergoing positive selection related to adaptive evolution. In conclusion, this study enriches the genomic data for *Hippophae* and provides valuable insights into the structural, evolutionary, and functional characteristics of plant mitochondrial genomes. These findings have important implications for the genetic improvement and conservation of *Hippophae* species.

## Data Availability

The chloroplast genome supporting this study has been deposited in GenBank (http://www.ncbi.nlm.nih.gov; accessed on 20 November 2024) under accession number PQ469773, and the mitochondrial genome under accession number PQ653489. Additionally, the HiFi sequencing data of *H. salicifolia* are available in the China National GeneBank Database under accession number CRA020057 and can be accessed at https://ngdc.cncb.ac.cn/gsa/browse/CRA020057/CRR1355215; accessed on 20 November 2024.

## Supplementary Materials

The following supporting information can be downloaded at: https://www.mdpi.com/article/10.3390/biology14040448/s1. Figure S1: Preliminary assembly draft of the mitochondrial genome of *H. salicifolia*. Figure S2: Distribution of lengths of interspersed repeats in the *H. salicifolia* mitogenome. Table S1: NCBI accession numbers of mitochondrial genomes of related species used in this study. Table S2: Functional classifications and physical locations of genes in the mitochondrial genome of *H. salicifolia*. Table S3: Distribution of SSRs in the mitochondrial genome of *H. salicifolia*. Table S4: Distribution of tandem repeats in the mitochondrial genome of *H. salicifolia*. Table S5: Distribution of dispersed repeats in the mitochondrial genome of *H. salicifolia*. Table S6: Codon usage of amino acid pairs in the mitochondrial genomes of four species. Table S7: Prediction of RNA editing sites in the mitochondrial genome of *H. salicifolia*. Table S8: The prediction of RNA editing sites in the mitogenome of *H. salicifolia*. Table S9: Homologous fragments between mitochondria and chloroplasts in *H. salicifolia*.
