# Supplementary material for: Assembly and Comparative Analysis of the Complete Mitochondrial Genome of Hippophae salicifolia
Source: Biology (Basel). 2025 Apr 20;14(4):448. doi: 10.3390/biology14040448 (PMC12025085; doi:10.3390/biology14040448)
Supplement: Supplementary file 1 [file biology-14-00448-s001.zip › Supplementary Material Figures.pdf]

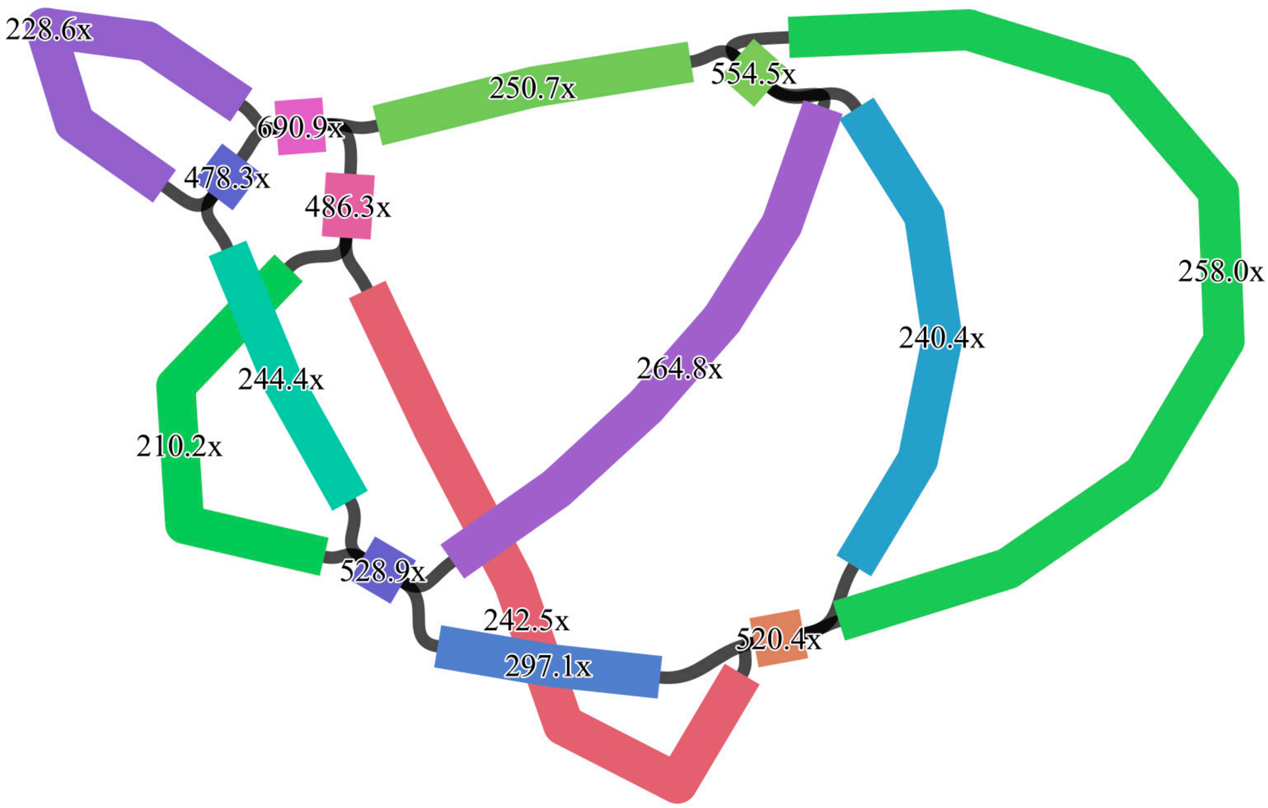

Figure S1. Preliminary assembly draft of the mitochondrial genome of *H. salicifolia*

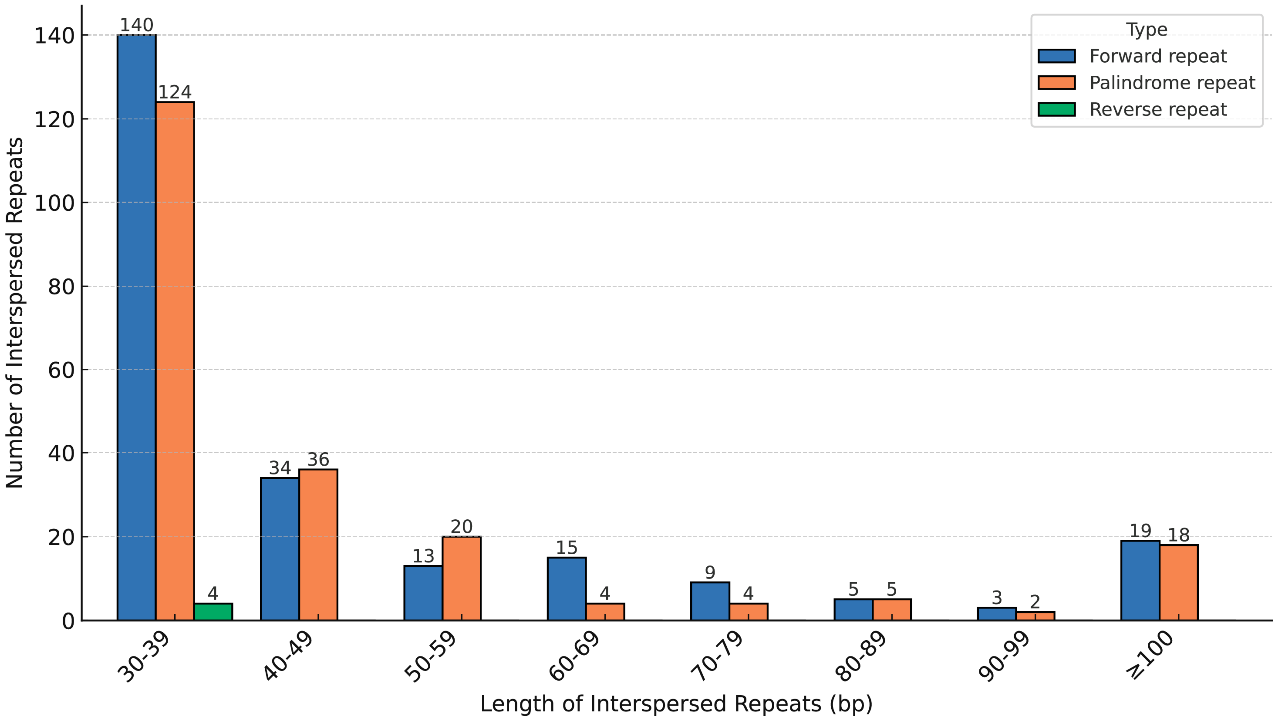

Figure S2. Distribution of lengths of interspersed repeats in the *H. salicifolia* mitogenome.
